# Tribenzoylarsine – A Benign Arsenic Precursor for InAs Nanocrystals

**DOI:** 10.1002/smll.202504624

**Published:** 2025-09-10

**Authors:** Artsiom Antanovich, Volodymyr Shamraienko, Jannika Lauth, Vladimir Lesnyak

**Affiliations:** ^1^ Institute of Physical Chemistry and Electrochemistry Leibniz University Hannover Callinstraße 3a 30167 Hannover Germany; ^2^ Physical Chemistry TU Dresden Zellescher Weg 19 01069 Dresden Germany; ^3^ Laboratory of Nano and Quantum Engineering Leibniz University Hannover Schneiderberg 39 30167 Hannover Germany; ^4^ Cluster of Excellence PhoenixD (Photonics Optics, and Engineering–Innovation Across Disciplines) Welfengarten 1a 30167 Hannover Germany; ^5^ Institute of Physical and Theoretical Chemistry University of Tübingen Auf der Morgenstelle 18 72076 Tübingen Germany

**Keywords:** acylarsine, indium arsenide, photoluminescence, quantum dots

## Abstract

III‐V semiconductor nanocrystals (NCs) have emerged as a benign alternative to II‐VI and IV‐VI NCs, which are restricted due to the toxicity of the comprising elements. While InP NCs advanced significantly, the development of infrared‐emitting InAs NCs has been relatively slow‐paced. This is due to the synthetic challenges arising from the highly covalent bonding in InAs and the limited range of available arsenic sources. Most importantly, current syntheses rely on hazardous pyrophoric and costly reagents, which hampers their wider adoption. In this work, the synthesis of tribenzoylarsine is reported, which can be used as a novel nonvolatile, nonpyrophoric, and hence safer precursor for metal arsenide nanoparticles. Tribenzoylarsine reacts with indium oleate without additional reducing agents yielding small (≈2 nm) InAs NCs. By tuning synthesis parameters (e.g., the addition of zinc oleate or using continuous injection) it is possible to cover the photoluminescence range of 630–780 nm with full width at half maximum as low as 170–210 meV, which is a very competitive value to the existing synthetic approaches. Along with the significantly safer nature of tribenzoylarsine and the great potential for synthesis optimization, this makes it a very promising alternative to the currently used arsenic precursors.

## Graphical abstract

A limited choice of available organoarsenic precursors and the need to employ hazardous reagents in the synthesis are among the main reasons behind the slow‐paced development of InAs nanocrystals. Here, the multigram synthesis of tribenzoylarsine – a novel nonpyrophoric and nonvolatile precursor is reported, which reacts with indium oleate without hazardous additives yielding small InAs nanocrystals.

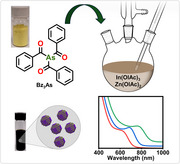

## 1 Introduction

Over the decades since their discovery, colloidal semiconductor nanocrystals (NCs) have emerged as important materials for a plethora of applications in multiple branches of technology. Various aspects of NCs have seen tremendous developments, spanning from the design of synthetic protocols and post‐synthetic processing to tailoring NCs for particular requirements and the construction of proof‐of‐concept devices.^[^ ^1^, ^2^, ^3^ ^]^

The major strides in the field have been made for II‐VI and IV‐VI semiconductor NCs, due to the broad availability of starting materials and relative simplicity of synthetic protocols for obtaining high‐quality NCs with varying shape, tunable composition, structure, and surface properties. However, recently, their attractiveness was severely undercut by tightening restrictions on the use of elements comprising such crystals, namely Cd, Pb, and Hg due to their toxicity.^[^ ^4^ ^]^ This highlights the importance of alternative materials, among which III‐V semiconductors stand out due to the relatively benign nature of the composing elements and improved chemical stability due to a higher covalent bonding nature.^[^ ^5^ ^]^ Although the first syntheses of III‐V colloidal NCs can be traced back to the early 90s,^[^ ^6^, ^7^, ^8^, ^9^ ^]^ their development was much slower than that of their II‐VI and IV‐VI counterparts. This is mainly due to the need of employing highly reactive pnictogen sources with limited commercial availability and markedly different reaction kinetics, which severely alters the relationship between nucleation and growth stages, preventing fine control over the NC size and size distribution.

Nevertheless, in the last 10 years, the field of III‐V NCs has experienced a dramatic increase in research interest and significant advances.^[^ ^10^ ^]^ At the same time, the success is mainly limited to InP NCs, covering the visible range of the electromagnetic spectrum, while infrared (IR)‐emitting NCs remain mainly represented by mercury and lead‐containing materials.^[^ ^10^, ^11^ ^]^ In this regard, InAs represents an attractive alternative due to its narrow bandgap (0.34–0.354 eV)^[^ ^11^, ^12^, ^13^ ^]^ and the large exciton Bohr radius (31–45 nm),^[^ ^13^, ^14^, ^15^ ^]^ suggesting broad tunability of optical properties deep into the IR region. However, the development of colloidal InAs NCs has been relatively slow‐paced, due to the aforementioned challenges of III‐V NCs colloidal syntheses exacerbated by severely limited choice of available arsenic sources.^[^ ^5^, ^10^ ^]^

Although some other arsenic precursors^[^ ^16^, ^17^, ^18^, ^19^, ^20^, ^21^, ^22^ ^]^ have been suggested, so far the range of sources employed in the syntheses is mainly restricted to two reagents, namely tris(trimethylsilyl)arsine (TMS_3_As)^[^ ^7^, ^23^, ^24^, ^25^ ^]^ and tris(dimethylamino)arsine (As(NMe_2_)_3_).^[^ ^26^, ^27^, ^28^, ^29^, ^30^, ^31^, ^32^, ^33^, ^34^, ^35^ ^]^ The most significant advances and the highest quality of InAs NCs were achieved with TMS_3_As, but it has several significant disadvantages such as high reactivity resulting in its fast depletion during the nucleation, which precludes further NC growth, as well as highly pyrophoric and hazardous nature, and high cost. At the same time, As(NMe_2_)_3_ offers more leverages to tune the reaction, thus potentially broadening the available wavelength range through employing various reducing agents,^[^ ^11^, ^29^, ^30^, ^31^, ^32^ ^]^ which are still usually pyrophoric, posing undesirable hazards for their broader and large‐scale adoption.

In this work, we report the preparation of a new arsenic precursor – tribenzoylarsine (Bz_3_As), and its performance in the colloidal synthesis of InAs NCs. This arsenic source can be easily and inexpensively synthesized from common starting reagents on a multigram scale, it is non‐pyrophoric and non‐volatile, which ensures safer and easier handling. In addition, we demonstrate that Bz_3_As reacts with indium oleate (or indium/zinc oleate) without the use of reducing agents to produce small InAs NCs exhibiting photoluminescence (PL) in the range of 630–780 nm with narrow full width at half maximum (FWHM) of 170–210 meV.

## 2 Results and Discussion

**Scheme 1:**
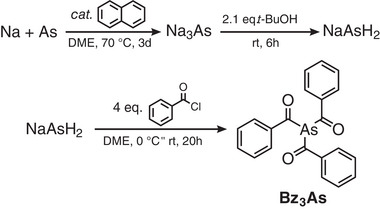
Scheme of Bz_3_As synthesis.

Tribenzoylarsine (Bz_3_As) was synthesized by adapting our recently reported procedure for acylphosphines (see Experimental Section).^[^ ^36^ ^]^ First, sodium arsenide was prepared in refluxing 1,2‐dimethoxyethane (DME) from respective elements in the presence of naphthalene acting as a catalyst (**Scheme** **1**). Afterward, it was activated by protonation with *tert*‐butanol at room temperature^[^ ^37^ ^]^ and finally reacted with the excess of benzoyl chloride.

Precipitation of crude Bz_3_As from tetrahydrofuran (THF) solution with hexane followed by drying in vacuo resulted in pale yellow powder with >50% overall yield. The purity and composition of the synthesized acylarsine were confirmed using ^1^H and ^13^C NMR (Figure S1, Supporting Information) and elemental analysis, which closely matched the expected results. Bz_3_As was found to be highly soluble in CHCl_3_ and THF, moderately soluble in triglyme, and only sparsely soluble in hexane. It is slightly air‐stable since it can be briefly subjected to air without a noticeable reaction. However, after ca. 10 min of exposure to normal conditions Bz_3_As becomes completely inactive and powder discoloration can be noticed, indicating the reaction of Bz_3_As with oxygen and/or water.

To examine the applicability of Bz_3_As for the synthesis of InAs NCs and perform preliminary optimization, we chose the heat‐up approach, due to its relative simplicity and general desirability for large‐scale procedures. First, we tested the reactivity of Bz_3_As toward indium carboxylates by injecting 0.6 mmol of Bz_3_As into 0.1 m solution of indium oleate (total amount 1 mmol) at 90 °C followed by heating the mixture at 190 °C for 60 min to ensure the complete conversion of precursors. Although higher temperatures (>240 °C) are generally considered to be necessary for the formation of high‐quality InAs NCs,^[^ ^11^ ^]^ we chose to limit the synthesis temperature based on the results of the preliminary experiments (see Figure S2 in the Supporting Information). They showed that although absorption and PL features improve up to ca. 220 °C, higher temperatures lead to significant broadening of optical features, which can be attributed to Ostwald ripening. This is also accompanied by severe NC aggregation and precipitation as well as InAs decomposition, which becomes apparent above 260 °C, when a minor amount of grey solid (most likely elemental arsenic) starts to deposit on the flask walls.

We also studied the effect of zinc carboxylates present in the reaction mixture on the properties of the resulting NCs, since zinc salts are known to alter the nucleation and growth of III‐V NCs and markedly reduce their spectral linewidths as well as improve PL quantum yield (QY).^[^ ^30^, ^38^, ^39^, ^40^ ^]^ The reactions were carried out in the same manner as described above except for the presence of zinc oleate in the reaction mixture yielding In:Zn ratio of 1:0.5 and 1:1.5.

**Figure 1:**
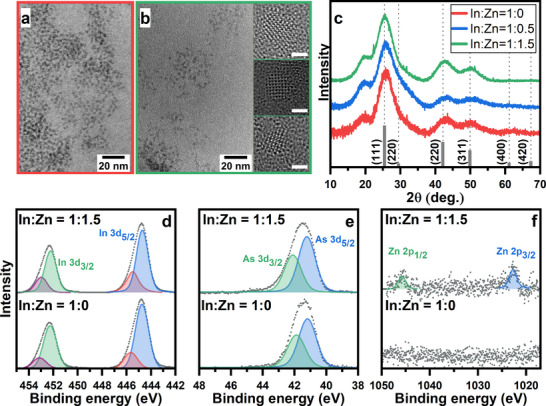
TEM images (a, b), XRD patterns (c) and XPS spectra (d‐f) of In(Zn)As NCs prepared without (a) and with the addition of zinc oleate (b). Dashed lines in (c) correspond to the XRD pattern of bulk zinc blende InAs and serve as a guide for the eye. Insets in (b) show high‐resolution TEM images of In(Zn)As NCs; scale bar is 1 nm.

Transmission electron microscopy (TEM) images in **Figure** **1**a,b show that InAs NCs synthesized both with and without zinc oleate are quite small ≈2 nm and have an irregular shape. Elemental analysis by inductively coupled plasma optical emission spectroscopy (ICP‐OES, **Table** **1**) reveals that although the reaction was conducted with nearly double the indium excess, the NCs formed are slightly arsenic‐rich, with an In:As ratio of 0.94. In the absence of other ligands, which can provide colloidal stabilization of NCs in the reaction mixture, this can be attributed to the fact that in addition to strongly bound X‐type oleate ligands the NC surface is covered by loosely bound Z‐type indium carboxylates, which can be relatively easily removed during excessive purification, especially in the presence of amines (or in our case pyridine, which was used to remove potential organic contaminants before the analyses).^[^ ^41^, ^42^, ^43^, ^44^ ^]^ This is further supported by the results of Choi et al.,^[^ ^45^ ^]^ demonstrating that displacement of Z‐type indium carboxylates by the ligand exchange results in the nearly stoichiometric InAs quantum dots (QDs). Elemental analysis also shows that In(Zn)As QDs are even richer in arsenic with an overall (In+Zn):As ratio dropping to 0.82. In addition, the zinc content in the NCs was found to be 1 and 11% for In:Zn feed ratios of 1:0.5 and 1:1.5, respectively.

**Table 1:** The composition of In(Zn)As QDs determined by ICP‐OES, lattice constants and lattice constant changes relative to bulk InAs (ε_bulk_) and InAs QDs prepared in Zn‐free conditions (ε_InAs_).

Sample	ICP‐OES		XRD		
	(In+Zn):As	Zn:In	a (Å)^a)^	ε_bulk_ (%)	ε_InAs_ (%)
**In:Zn = 1:0**	0.94 ± 0.013	0.00	5.98	−1.2%	‐
**In:Zn = 1:0.5**	0.82± 0.048	0.01	5.98	−1.2%	0%
**In:Zn = 1:1.5**	0.82± 0.015	0.11	6.02	−0.7%	+ 0.5%

^a)^ Average from positions of (111), (220), and (311) reflections.

The X‐Ray diffraction (XRD) patterns (Figure 1c) of the InAs QDs exhibit reflections characteristic for cubic zinc blende InAs, which are significantly broadened due to the small NC size and slightly shifted to larger 2θ angles corresponding to 1.2% lattice contraction (Table 1) relative to bulk InAs (a = 6.0583 Å)^[^ ^12^ ^]^. At the same time, XRD patterns of In(Zn)As NCs are nearly identical to those of InAs NCs prepared without zinc oleate. Regardless of the relatively high zinc content, the reflections are negligibly shifted to lower angles, as was previously seen in Zn‐doped InAs,^[^ ^46^ ^]^ pointing to the lack of significant In‐to‐Zn substitution and the localization of Zn atoms near the surface or in interstitial positions.^[^ ^30^, ^47^ ^]^ Based on the width of the diffraction peaks with the Scherrer equation we estimated the NC size to be ≈1.6 nm, which agrees quite well with the TEM measurements, suggesting that even relatively low‐temperature synthesis yields InAs NCs with good crystallinity,^[^ ^28^ ^]^ and is supported by the high‐resolution TEM images, exhibiting pronounced lattice fringes (inset in Figure 1b).

Aside from the reflections typical for indium arsenide, diffractograms exhibit a broad shoulder at ≈20°. This feature is frequently observed in nanoparticle diffractograms and was assigned to “forbidden” reflection due to lattice distortions,^[^ ^48^, ^49^ ^]^ remaining precursors,^[^ ^50^ ^]^ or ligand shell ordering.^[^ ^51^ ^]^ To further exclude the presence of other phases in our samples, we performed X‐ray photoelectron spectroscopy (XPS) measurements (Figure 1d–f). The XPS spectra in the In 3d region (Figure 1d) exhibit two intensive peaks at 444.7 and 452.1 eV, which are assigned to In 3d_5/2_ and In 3d_3/2_ levels in InAs.^[^ ^22^ ^]^ Additionally, low‐intensity shoulders at slightly higher energies are observed, which can be attributed to the surface In atoms bound with X‐type carboxylates.^[^ ^45^ ^]^ Spectra in the arsenic region (Figure 1e) reveal a single broad band centered at 41.3 eV, comprising of two overlapping As 3d_3/2_ and As 3d_5/2_ peaks. Notably, no signals of As‐O species, which are generally situated at ≈44 eV, are observed, thus excluding surface oxidation. The incorporation of zinc ions into NCs is further supported by the XPS data, showing the emergence of two peaks in the Zn 2p region (Figure 1f) at 1045 and 1022 eV in line with previous observations.^[^ ^22^ ^]^ Additionally, no significant shifts in the XPS spectra in the In 3d region occur, which further supports the lack of extensive Zn‐to‐In substitution.^[^ ^47^ ^]^

Interestingly, considering the structure of Bz_3_As and the fact that arsenic has a lower electronegativity than carbon (2.211 vs. 2.544, respectively),^[^ ^52^ ^]^ one can assume that the arsenic atom bears a positive charge and hence, similarly to As(NMe_2_)_3_, the addition of a reducing agent should be required for the reaction. Nevertheless, the reaction proceeds without extra additives. While the determination of the exact reaction pathway requires more sophisticated investigations, some potential mechanisms (or their combination) can be inferred to account for the formation of InAs NCs.

**Scheme 2:**
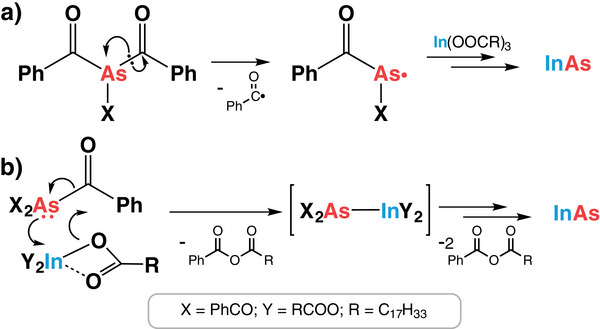
Potential mechanisms of the reaction of Bz_3_As with indium carboxylate.

The first one involves the formation of radical species stabilized by the delocalization involving carbonyl and phenyl groups, considering that some acyl metalloids are prone to homolytic metalloid‐carbon bond cleavage (**Scheme** **2**a).^[^ ^53^ ^]^ This mechanism, however, is considered very unlikely due to the high probability of unspecific side reactions in a complex reaction mixture. A more realistic scenario involves coordination of indium atoms by the arsenic lone pair, followed by the intramolecular rearrangement resembling a Friedel‐Crafts acylation mechanism (Scheme 2b), which is known to be catalyzed by Lewis acids, including indium compounds.^[^ ^54^, ^55^ ^]^ A similar intramolecular reaction was shown to be the underlying reason for the formation of nickel phosphides using organophosphites, containing phosphorus atoms in a +3 oxidation state, without the external addition of reducing agents.^[^ ^56^ ^]^ Moreover, in a recent report^[^ ^57^ ^]^ the acylation mechanism was demonstrated in a reaction of analogous acylphosphines with indium compounds yielding InP NCs.

To monitor the evolution of InAs NC optical properties, aliquots were withdrawn from the mixture during the reaction. In addition, the sample absorption at 400 nm was monitored to estimate the arsenic source conversion rate using the approach from refs. [27, 39, 58]. It is worth noting that this approach relies on the assumption that the intrinsic absorption coefficient of NCs in this region is very close to that of the bulk material.^[^ ^59^ ^]^ However, for very small NCs this might result in overestimated values due to the contribution of quantum confinement but still allows for semiquantitative analysis.

**Figure 2:**
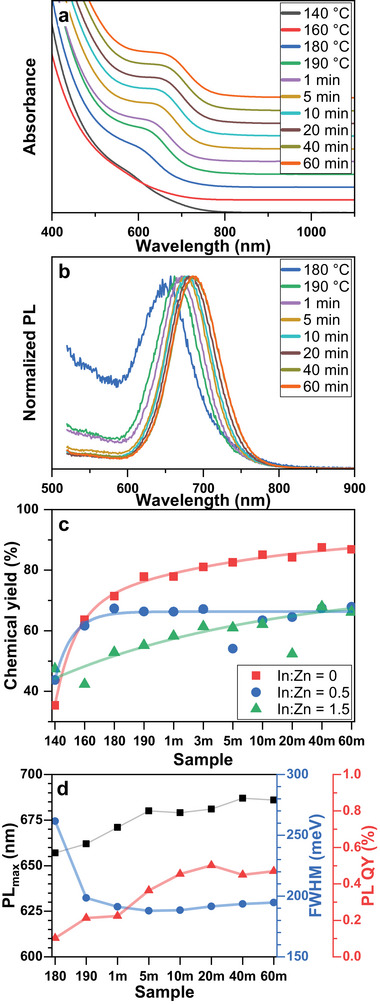
Evolution of the absorption (a), PL (b) spectra, chemical yield determined from the sample absorbance at 400 nm (c), and spectral parameters (d) during the heat‐up single‐injection synthesis of InAs NCs.

After the Bz_3_As injection, the reaction mixture becomes deep red presumably due to the formation of decomposition products of Bz_3_As and amorphous InAs “clusters”. Upon further temperature increase above 160 °C, a shoulder at ≈600 nm emerges in the absorption spectra accompanied by the appearance of a weak PL signal pointing to the formation of InAs NCs (**Figure** **2**a,b). Upon further heating, the NCs continue to form as evidenced by slowly increasing chemical reaction yield calculated based on sample absorption and reaching ca. 80% (Figure 2c) at approx. 5 min. Such behavior differs from the reported nearly instantaneous conversion of TMS_3_As^[^ ^21^, ^24^ ^]^ or slow As(NMe_2_)_3_ conversion over tens of minutes depending on the reducing agents,^[^ ^27^, ^30^ ^]^ thus allocating Bz_3_As in between these arsenic sources on the reactivity scale. To further validate this claim, we conducted an additional experiment using TMS_3_As as an arsenic source in identical conditions. After the injection at 90 °C, the reaction mixture instantaneously becomes black. The absorption spectra (Figure S3 in the Supporting Information) exhibit a very broad featureless shoulder indicating the formation of a mixture of polydisperse NCs. Unlike in the case of Bz_3_As, demonstrating steady redshift and sharpening of excitonic bands, the absorption spectra remain practically unchanged until later stages of reaction, indicating fast and complete depletion of the precursor during the injection. Only after prolonged heating at 190 °C (>20 min) a poorly pronounced absorption feature starts to emerge, due to size‐focusing through the Ostwald ripening of the polydisperse NC mixture.

The redshift of PL follows absorption spectra in the same timeframe covering the range of 660–685 nm, while PL FWHM drops below 190 meV (Figure 2d). Together with the chemical yield data, this indicates that InAs NC growth mainly occurs through precursor conversion resulting in the size‐dependent growth of III‐V NCs,^[^ ^60^, ^61^ ^]^ rather than NC Ostwald ripening. At the same time, the PL QY continued to rise to approx. 0.5%, which can be attributed to further NC crystallization and improved surface passivation by the ligands.

**Figure 3:**
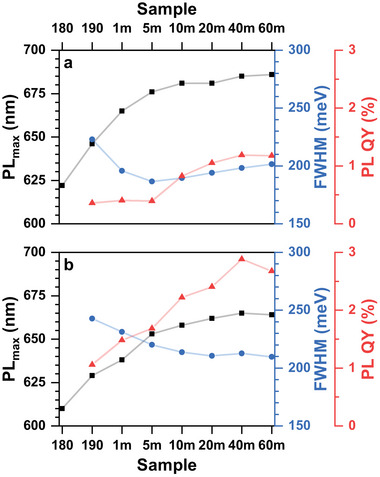
Evolution of the spectral parameters during the heat‐up single‐injection synthesis of InAs NCs with the addition of 0.5 (a) and 1.5 (b) mmol of zinc oleate.

While in the presence of zinc oleate the evolution of InAs NC optical properties is qualitatively similar to the one described above (**Figures** **3** and S4 in the Supporting Information), closer examination reveals some differences in the reaction progress. The analysis of sample absorbance at 400 nm demonstrates that the addition of zinc oleate results in a lower overall reaction yield of ca. 65% for both In:Zn feed ratios (Figure 2c). Besides, the InAs NC formation rate is noticeably slower, which is especially apparent at higher zinc concentration. This can be potentially attributed to the combination of i) the competition of indium and zinc carboxylates for complexation with Bz_3_As at the start of the reaction (see Scheme 2b), contributing to the precursor loss in side reactions, and ii) the increased NC surface passivation due to higher metal oleate concentration, resulting in a denser ligand shell and hindered NC growth via monomer addition.

Changes in the PL spectra positions are likewise slowed down, resulting in a slightly broader PL coverage of 620–685 nm and 610–665 nm achieved with a In:Zn feed ratio of 1:0.5 and 1:1.5, respectively. This is in line with TEM and XRD data considering that a slight PL blue shift with a higher Zn feed amount can be assigned to the higher Zn incorporation rate. Similarly to the Zn‐free synthesis, the PL QY of the In(Zn)As NCs gradually improves over the course of the reaction and reaches noticeably higher values of 1.2 and 2.9% in the case of the In:Zn feed ratio of 1:0.5 and 1:1.5, respectively, which is in line with improved surface passivation under Zn‐rich conditions.^[^ ^22^, ^30^ ^]^ At the same time, the PL FWHM values of samples with different zinc feed ratios follow a slightly different trend. At lower zinc concentration (In:Zn = 1:0.5) it is similar to the Zn‐free synthesis, i.e. reaches the minimum of 186 meV and slowly increases to 201 meV, suggesting that slow Ostwald ripening occurs. The PL FWHM of the InAs sample with the highest zinc loading gradually decreases over the whole reaction time reaching 209 meV by benefiting from the slower reaction and supply of monomers regulating the size‐dependent growth of the InAs NCs.

Although the addition of zinc oleate slows down the InAs growth resulting in a slightly wider accessible PL range, the attained NC size range remains narrow compared to the one achieved in the synthesis of II‐VI and IV‐VI NCs. This is a known problem of the synthesis of III‐V semiconductor NCs, stemming from the rapid consumption of the pnictogen precursors during the nucleation stage, which leads to a low concentration of the remaining precursor and in turn precludes the “focusing” of the size distribution as well as growing larger particles through monomer addition.^[^ ^62^ ^]^ Furthermore, the growth kinetics of III‐V NCs is distinct from II‐VI and IV‐VI NCs and is barely affected by the regulation of the reactivity of pnictogen precursors.^[^ ^21^, ^62^, ^63^, ^64^ ^]^ Alternatively, this challenge can be overcome by a continuous slow injection of either As‐precursors or InAs “clusters” after NC nucleation.^[^ ^25^, ^61^, ^65^, ^66^ ^]^

In a similar fashion, to expand the size range of InAs NCs, in another set of experiments, the total amount of 0.6 mmol of Bz_3_As was divided into two unequal portions (see Experimental section for more details). One part was swiftly injected into the flask to induce nucleation, whereas the remaining part was slowly added dropwise via a syringe as a 0.1 M solution in triglyme at a 2 mL/h rate to supply the As‐precursor for further growth of InAs NCs. We tested two different ratios: for example, to prepare samples labeled S02/04, 0.2 mmol of Bz_3_As was quickly injected, whereas the remaining 0.4 mmol were used for slow addition. Samples labeled S04/02 were prepared in a similar manner, except for 0.4 mmol of the arsenic source were injected first followed by the slow addition of the remaining 0.2 mmol.

**Figure 4:**
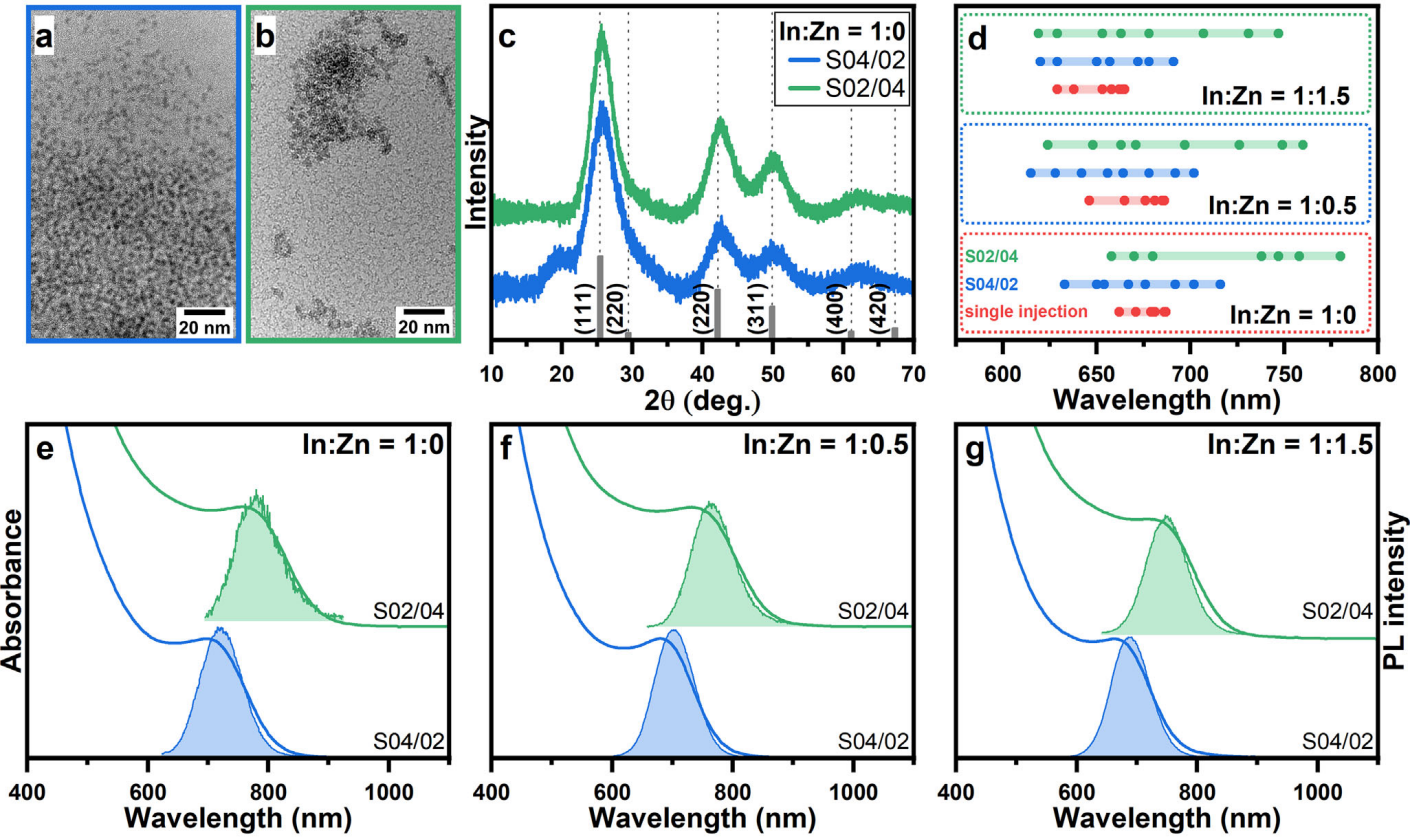
TEM images (a, b) and XRD patterns (c) of InAs NCs prepared with the additional portion of 0.2 (a) and 0.4 (b) mmol of Bz_3_As. Optical range of PL maxima of In(Zn)As NCs, depending on the synthesis conditions (d). The points on the chart are the positions of PL maxima from spectra presented in Figure 1 and Figures S4–S7 (Supporting Information). Absorption and PL spectra of In(Zn)As NCs prepared via the additional slow injection without (e) and with the addition of 0.5 (f) and 1.5 (g) mmol of zinc oleate.

As can be seen from **Figure** **4**a,b, the average InAs NC size increased to ca. 2.7 ± 0.37 nm and 2.9 ± 0.48 nm for S04/02 and S02/04 samples, respectively. It is also noticeable that with the size increase the shape starts to deviate from quasi‐spherical to elongated, which is typical for slow growth of InAs NCs in concentrated solutions.^[^ ^25^ ^]^ At the same time, the XRD patterns (Figure 4c) become narrower corresponding to a Scherrer size of 1.9 nm and 2.3 nm, respectively, without experiencing any noticeable shifts even under zinc‐rich conditions (Figure S5 and Table S1 in the Supporting Information). Additionally, the intensity of the feature at ca. 20° relative to (111) reflection significantly decreases with the increase of the QD size, which aligns well with the observations in ref.[51] and further supports its assignment to the organic ligand shell.

According to ICP‐OES results, compared to particles prepared via the single injection the metal:arsenic ratio rises as the particle size increases (**Table** **2**). In addition, the samples prepared with In:Zn feed ratio of 1:0.5 and 1:1.5 demonstrate the same (1%) or slightly lower (8%) degree of zinc incorporation. Both these observations can be attributed to the decrease of the surface‐to‐volume ratio and hence the relative amount of weakly‐bound metal oleates.

**Table 2:** The composition of In(Zn)As QDs prepared by a continuous injection determined by ICP‐OES.

Sample		(In+Zn):As	Zn:In
**In:Zn = 1:0**	S04/02	0.99 ± 0.015	0.00
	S02/04	1.04 ± 0.014	0.00
**In:Zn = 1:0.5**	S04/02	1.22 ± 0.005	0.01
	S02/04	1.01 ± 0.009	0.01
**In:Zn = 1:1.5**	S04/02	0.93 ± 0.016	0.10
	S02/04	0.96 ± 0.015	0.08

Upon the slow addition of the As‐precursor, the absorption bands of InAs NCs steadily shift to lower energies, covering a broader wavelength range (Figures S6 and S7 in the Supporting Information). Similarly, the slow addition expands the accessible range of PL spectra of zinc‐free samples to approx. 630–730 nm and 630–780 nm for S04/02 and S02/04 samples, respectively (Figure 4d). The samples prepared with extra zinc oleate cover a similar range albeit exhibiting a slight blue shift with increasing the Zn feed concentration. Aside from widening the attainable InAs NC size range, the continuous injection allowed for the improvement of the particle size distribution, as evidenced by the narrower PL FWHM reaching 170 meV and absorption bands becoming more pronounced with the best‐achieved peak‐to‐value ratio of 1.08 (Figure 4e–g). This agrees well with previous reports on III‐V NCs^[^ ^61^, ^65^, ^67^ ^]^ and highlights the importance of moderating the monomer supply, e.g. by decoupling the precursor conversion and NC nucleation and growth.

## 3 Conclusion

In this work, we report the synthesis of tribenzoylarsine – a novel arsenic precursor for metal arsenide NCs, which can be prepared from inexpensive starting materials with high yield and purity. It can be considered a much safer alternative to commonly used arsenic sources, such as tris(trimethylsilyl)arsine and tris(dimethylamino)arsine, due to its nonvolatile and nonpyrophoric nature. In addition, we demonstrate that by heating tribenzoylarsine with indium oleate small InAs NCs exhibiting PL at ca. 670 nm with FWHM of 186 nm can be synthesized. We show that by adding zinc oleate or by slow secondary addition of arsenic source, one can alter the reaction kinetics resulting in an expanded InAs NC PL range of 620–780 nm and improved PL FWHM down to 170 meV. Although the PL FWHM values achieved in this work are larger than in the state‐of‐the‐art InAs QDs prepared with TMS_3_As, they are well within the range reported to date.^[^ ^11^ ^]^ Considering that existing procedures have been improved for more than two decades, and a wide parameter space is yet to be investigated, we believe that Bz_3_As is a very promising alternative to the currently used arsenic sources, especially considering its better safety profile.

## 4 Experimental Section

### Materials

Arsenic (As, 99%, ‐20 mesh powder) was purchased from Strem. Benzoyl chloride (99.5+%), 1,2‐dimethoxyethane (DME, 99+%, stabilized with dibutylhydroxytoluene), hexane (97%, extra dry), sodium (≥99.8%), *tert*‐butanol (*t*‐BuOH, 99.5%), tetrahydrofuran (THF, 99.5%, stabilized, extra dry over molecular sieves), triethylene glycol dimethyl ether (triglyme, 99%, stabilized) were purchased from Acros. Indium (III) acetate (99.99%) was purchased from Alfa Aesar. Hydrogen peroxide (30%, trace metal grade), naphthalene (≥99%), 1‐octadecene (ODE, technical grade, 90%), zinc acetate dihydrate (Zn(OAc)_2_·2H_2_O, ≥99.0%) and standard solutions for inductively coupled plasma optical emission spectroscopy (ICP‐OES) determination of In, Zn, and As were purchased from Sigma‐Aldrich. Nitric acid (≥68%, trace metal grade) and oleic acid (OlAc, technical grade, 90%) were purchased from Fischer Chemicals. Deuterochloroform (99.8 at.% D, stabilized with Ag) was purchased from Carl Roth. (TMSi)_3_As was prepared according to ref.[64]. DME was dried by refluxing over sodium/benzophenone for 2 h followed by distillation under argon.^[^ ^68^ ^]^ Triglyme was degassed under vacuum (0.1 mbar) at room temperature for 5 h. Both solvents were stored in the nitrogen‐filled glovebox over 3 Å molecular sieves.

### Synthesis of Tribenzoylarsine (Bz_3_As)

***Caution!*** *Synthesis of sodium arsenide involves heating highly reactive compounds in a flammable solvent, which can pose a fire hazard. In addition, sodium arsenide is hydrolyzed by air moisture with the release of highly toxic arsine gas*.

Prior to use, all glassware was dried at 150 °C for 24 h. All operations were performed either in the nitrogen‐filled glovebox or on the Schlenk line under an argon atmosphere.

7.5 g (100 mmol) of As, 2.56 g (20 mmol) of naphthalene, and 80 mL of dry DME were mixed in a 500 mL three‐neck flask in the glovebox. The flask was removed from the glovebox and connected to the Schlenk line and a reflux condenser under an argon flow. Then, 8.1 g (350 mmol) of sodium cut into small chunks were added into the flask under argon flow. The flask was sealed with silicone septa, heated to 80 °C, and kept at this temperature for 3 days. Over the course of the reaction, the mixture in the flask turned from dark green to black with a suspended precipitate of sodium arsenide. After 3 days, the flask was cooled to room temperature, the reaction mixture was diluted with 150 mL of dry DME and a solution of 21.2 mL (210 mmol) of *t*‐BuOH in 30 mL of DME was slowly added via a syringe pump. After the addition, the mixture was vigorously stirred at room temperature for 6 h. Then, the flask was placed on an ice bath and 46.5 mL (400 mmol) of degassed benzoyl chloride were slowly added dropwise. Afterward, the ice bath was removed, and the reaction mixture was left stirring at room temperature for 20 h. During this time, it became slightly yellow with a significant amount of grey precipitate. ***Note***: *If the reaction mixture becomes too thick to stir, it can be further diluted with dry DME*. Subsequently, the solids were removed from the reaction mixture by centrifugation under argon yielding a yellow solution. All volatile compounds were removed from the supernatant under vacuum resulting in a pale‐yellow solid. The solid was dissolved under an inert atmosphere in a minimal amount of dry THF (ca. 80 mL) and to this solution, an excess (ca. 300 mL) of dry hexane was added causing the product to precipitate. The solid was isolated by centrifugation in tightly sealed tubes under argon. The product was further purified by another dissolution/precipitation cycle as described above. Finally, the isolated solid product was dried under vacuum at room temperature for 8 h and stored in the glovebox. Yield 22.6 g (58 %).

**^1^H NMR** (600 MHz, CDCl_3_): δ 7.95 (m, 6H, *o*‐H), 7.59 (m, 3H, *p*‐H), 7.49 (m, 6H, *m*‐H).

**^13^C NMR** (151 MHz, CDCl_3_): δ 210.6 (*C* = O), 140.3 (*C_ipso_*), 134.3 (*C_para_*), 129.2 (*C_arom_*), 129.1 (*C_arom_*).

Elemental analysis: calculated for C_21_H_15_O_3_As: C, 64.63; H 3.87; found (CHNS): C, 64.65 ± 0.01; H 3.70 ± 0.01. As: calculated 19.18; found (ICP‐OES): 18.2 ± 3.6.

### Indium and Indium/Zinc Oleate Stock Solutions

292 mg (1 mmol) of indium acetate, 950 µL (3 mmol) of OlAc, and 10 mL of ODE were mixed in a 50 mL three‐neck flask and heated up to 180 °C under an argon flow for 1 h to form indium oleate. Then, the temperature was lowered to 90 °C and the mixture was degassed for 1 h to remove volatile species and the flask was purged with argon.

Indium/zinc oleate stock solutions were prepared in the same manner except the flask was additionally loaded with 110 mg (0.5 mmol) of Zn(OAc)_2_·2H_2_O and 315 µL (1 mmol) of OlAc (In:Zn = 1:0.5) or 330 mg (1.5 mmol) of Zn(OAc)_2_·2H_2_O and 950 µL (3 mmol) of OlAc (In:Zn = 1:1.5).

### Synthesis of InAs and In(Zn)As NCs via Single Injection

234 mg (0.6 mmol) of Bz_3_As dispersed in 1 mL of degassed triglyme was injected into the flask containing metal oleates at 90 °C. The reaction mixture was heated up to 190 °C and kept for 1 h at this temperature.

### Synthesis of InAs and In(Zn)As NCs via Continuous Injection

To prepare samples S04/02, 156 mg (0.4 mmol) of Bz_3_As dispersed in 1 mL of triglyme were injected into the flask containing metal oleates at 90 °C. Shortly after the injection, the slow addition (2 mL/h) of 2 mL of 0.1 M triglyme Bz_3_As solution was started, and the flask was set to heat to 190 °C. After the addition, the reaction mixture was kept at this temperature for 30 min. Samples S02/04 were prepared in a similar manner, except 78 mg (0.2 mmol) were quickly injected into the metal precursor solution, followed by the slow addition of 4 mL of 0.1 M triglyme Bz_3_As solution.

For NC purification, the reaction mixture was allowed to cool to room temperature and brought into a nitrogen‐filled glovebox. First, NCs were precipitated with anhydrous ethanol and isolated by centrifugation. Purification was repeated two times using chloroform and ethanol as a solvent and nonsolvent, respectively.

For characterization, NCs were further purified by dissolving in 10 mL of anhydrous chloroform and 1 mL of degassed pyridine followed by adding the excess of anhydrous ethanol and centrifugation. Then two more precipitation cycles were performed using chloroform and methanol.

### Characterization

^1^H and ^13^C NMR spectroscopy was performed using a Bruker Avance III 600 spectrometer. For the measurements, Bz_3_As was dissolved in deuterochloroform (ca. 5 w/v %) in the nitrogen‐filled glovebox and transferred into air‐tight NMR tubes. Elemental analysis (CHNS) was done with FlashSmart Elemental Analyzer.

Absorption spectra were acquired using a UV−vis−NIR spectrophotometer Cary 5000 (Varian), and PL spectra were recorded on a Fluorolog‐3 spectrofluorometer (Horiba Jobin Yvon Inc.). PL QYs were determined by a reference method^[^ ^69^ ^]^ using oxazine‐1 in ethanol (QY = 0.141)^[^ ^70^ ^]^ as a comparison standard. Bright‐field TEM imaging was performed on a FEI Tecnai G2 F20 microscope equipped with a field emission gun operated at 200 kV. High‐resolution TEM images were acquired using a JEOL ARM200F equipped with a CEOS aberration corrector operating at 200 kV. Samples were prepared by dropcasting diluted NC solutions in chloroform onto 300 mesh copper grids coated with graphene oxide on holey carbon (EM Resolutions).

Powder XRD patterns were acquired using a Bruker D2 Phaser with a Cu source operated at 30 kV and 10 mA. Concentrated solutions of NCs in chloroform were dropcast on a Si wafer and dried under the air. XPS measurements were performed using VersaProbe III from Physical Electronics (ULVAC‐PHI) with an Al Kα X‐ray source (1486.6 eV at 25.2 W). Samples were prepared by dropcasting concentrated chloroform solutions onto Si wafers and drying in a nitrogen‐filled glovebox. Spectra were processed using CasaXPS software and charge corrected relative to C 1s signal at 284.8 eV.

For ICP‐OES measurements, aliquots of chloroform solutions of Bz_3_As (400 µL, 5 g L^−1^) or In(Zn)As NCs were transferred into a centrifugation tube and dried under a stream of nitrogen. Then the solids were decomposed for 48 h using 30% trace metal grade hydrogen peroxide and concentrated trace metal grade nitric acid according to the protocol from ref.[71]. Shortly before the measurements, the samples were diluted to 15 mL with Milli‐Q water. Elemental analysis was carried out in triplicate for each sample using an iCAP 7000 Series (Thermo Scientific).

## Conflict of Interest

The authors declare no conflict of interest.

## Supporting information



Supporting Information

## Data Availability

The data that support the findings of this study are available from the corresponding author upon reasonable request.

## References

[1] M. V. Kovalenko , L. Manna , A. Cabot , Z. Hens , D. V. Talapin , C. R. Kagan , V. I. Klimov , A. L. Rogach , P. Reiss , D. J. Milliron , P. Guyot‐Sionnest , G. Konstantatos , W. J. Parak , T. Hyeon , B. A. Korgel , C. B. Murray , W. Heiss , P. N. Nanocrystals , ACS Nano 2015, 9, 1012. 25608730 10.1021/nn506223h

[2] A. L. Efros , L. E. Brus , ACS Nano 2021, 15, 6192. 33830732 10.1021/acsnano.1c01399

[3] F. P. García de Arquer , D. V. Talapin , V. I. Klimov , Y. Arakawa , M. Bayer , E. H. Sargent , Science 2021, 373, aaz8541. 10.1126/science.aaz8541 34353926

[4] C. Campalani , J.‐C. M. Monbaliu , Mater. Sci. Eng. R Rep. 2025, 163, 100940.

[5] T. Kim , D. Shin , M. Kim , H. Kim , E. Cho , M. Choi , J. Kim , E. Jang , S. Jeong , ACS Energy Lett. 2023, 8, 447.

[6] R. L. Wells , S. R. Aubuchon , S. S. Kher , M. S. Lube , P. S. White , Chem. Mater. 1995, 7, 793.

[7] A. A. Guzelian , U. Banin , A. V. Kadavanich , X. Peng , A. P. Alivisatos , Appl. Phys. Lett. 1996, 69, 1432.

[8] H. Uchida , T. Matsunaga , H. Yoneyama , T. Sakata , H. Mori , T. Sasaki , Chem. Mater. 1993, 5, 716.

[9] O. I. Micic , C. J. Curtis , K. M. Jones , J. R. Sprague , A. J. Nozik , J. Phys. Chem. 1994, 98, 4966.

[10] T. A. Gazis , A. J. Cartlidge , P. D. Matthews , J. Mater. Chem. C 2023, 11, 3926.

[11] H. B Jalali , L. De Trizio , L. Manna , F. Di Stasio , Chem. Soc. Rev. 2022, 51, 9861. 36408788 10.1039/d2cs00490a PMC9743785

[12] O. Madelung , Semiconductors: Data Handbook, Semiconductors: Data Handbook, Springer Berlin Heidelberg, Berlin, Heidelberg, 2004.

[13] F. Wang , H. Yu , S. Jeong , J. M. Pietryga , J. A. Hollingsworth , P. C. Gibbons , W. E. Buhro , ACS Nano 2008, 2, 1903. 19206431 10.1021/nn800356z

[14] T. Puangmali , M. Califano , P. Harrison , J. Phys. Chem. C 2010, 114, 6901.

[15] T.‐G. Kim , D. Zherebetskyy , Y. Bekenstein , M. H. Oh , L.‐W. Wang , E. Jang , A. P. Alivisatos , ACS Nano 2018, 12, 11529. 30335943 10.1021/acsnano.8b06692

[16] J. Zhang , D. Zhang , CrystEngComm 2010, 12, 591.

[17] J. Lauth , T. Strupeit , A. Kornowski , H. Weller , Chem. Mater. 2013, 25, 1377.

[18] A. Das , A. Shamirian , P. T. Snee , Chem. Mater. 2016, 28, 4058.

[19] R. Tietze , R. Panzer , T. Starzynski , C. Guhrenz , F. Frenzel , C. Würth , U. Resch‐Genger , J. J. Weigand , A. Eychmüller , Part. Part. Syst. Charact. 2018, 35, 1800175.

[20] T. Zhao , N. Oh , D. Jishkariani , M. Zhang , H. Wang , N. Li , J. D. Lee , C. Zeng , M. Muduli , H.‐J. Choi , D. Su , C. B. Murray , C. R. Kagan , J. Am. Chem. Soc. 2019, 141, 15145. 31496238 10.1021/jacs.9b06652

[21] D. K. Harris , M. G. Bawendi , J. Am. Chem. Soc. 2012, 134, 20211. 23228014 10.1021/ja309863n PMC3535303

[22] S. Kim , S. Yeon , M. Lee , J. Jin , S. Shin , N. Gwak , I. Jeong , H. Jang , G. W. Hwang , N. Oh , NPG Asia Mater. 2023, 15, 30.

[23] D. Battaglia , X. Peng , Nano Lett. 2002, 2, 1027. 10.1021/nl062336y 17297994

[24] R. Xie , X. Peng , Angew. Chem., Int. Ed. 2008, 47, 7677. 10.1002/anie.200802867 18756560

[25] T. Kim , S. Park , S. Jeong , Nat. Commun. 2021, 12, 3013. 34021149 10.1038/s41467-021-23259-w PMC8140152

[26] M. Green , S. Norager , P. Moriarty , M. Motevalli , P. O'Brien , J. Mater. Chem. 2000, 10, 1939.

[27] V. Grigel , D. Dupont , K. De Nolf , Z. Hens , M. D. Tessier , J. Am. Chem. Soc. 2016, 138, 13485. 27701864 10.1021/jacs.6b07533

[28] V. Srivastava , E. M. Janke , B. T. Diroll , R. D. Schaller , D. V. Talapin , Chem. Mater. 2016, 28, 6797.

[29] V. Srivastava , E. Dunietz , V. Kamysbayev , J. S. Anderson , D. V. Talapin , Chem. Mater. 2018, 30, 3623.

[30] D. Zhu , F. Bellato , H. Bahmani Jalali , F. Di Stasio , M. Prato , Y. P. Ivanov , G. Divitini , I. Infante , L. De Trizio , L. Manna , J. Am. Chem. Soc. 2022, 144, 10515. 35648676 10.1021/jacs.2c02994 PMC9204758

[31] M. Ginterseder , D. Franke , C. F. Perkinson , L. Wang , E. C. Hansen , M. G. Bawendi , J. Am. Chem. Soc. 2020, 142, 4088. 32073841 10.1021/jacs.9b12350

[32] M. Kim , J. Lee , J. Jung , D. Shin , J. Kim , E. Cho , Y. Xing , H. Jeong , S. Park , S. H. Oh , Y.‐H. Kim , S. Jeong , J. Am. Chem. Soc. 2024, 146, 10251. 38587307 10.1021/jacs.4c00966 PMC11027140

[33] J. Leemans , K. C. Dümbgen , M. M. Minjauw , Q. Zhao , A. Vantomme , I. Infante , C. Detavernier , Z. Hens , J. Am. Chem. Soc. 2021, 143, 4290. 33710882 10.1021/jacs.0c12871

[34] J. Leemans , V. Pejović , E. Georgitzikis , M. Minjauw , A. B. Siddik , Y. Deng , Y. Kuang , G. Roelkens , C. Detavernier , I. Lieberman , P. E. Malinowski , D. Cheyns , Z. Hens , Adv. Sci. 2022, 9, 2200844. 10.1002/advs.202200844 PMC9189642 35398996

[35] J. Leemans , D. Respekta , J. Bai , S. Braeuer , F. Vanhaecke , Z. Hens , ACS Nano 2023, 17, 20002. 37787479 10.1021/acsnano.3c05138

[36] A. Antanovich , A. Iodchik , J. Li , P. Khavlyuk , V. Shamraienko , V. Lesnyak , Small 2025, 21, 2409389. 39703038 10.1002/smll.202409389 PMC11817936

[37] A. Hinz , J. M. Goicoechea , Angew. Chem., Int. Ed. 2016, 55, 8536. 10.1002/anie.201602310 PMC5074235 27093942

[38] S. Koh , T. Eom , W. D. Kim , K. Lee , D. Lee , Y. K. Lee , H. Kim , W. K. Bae , D. C. Lee , Chem. Mater. 2017, 29, 6346.

[39] M. D. Tessier , D. Dupont , K. De Nolf , J. De Roo , Z. Hens , Chem. Mater. 2015, 27, 4893.

[40] W.‐S. Song , H.‐S. Lee , J. C. Lee , D. S. Jang , Y. Choi , M. Choi , H. Yang , J. Nanoparticle Res. 2013, 15, 1750.

[41] N. C. Anderson , M. P. Hendricks , J. J. Choi , J. S. Owen , J. Am. Chem. Soc. 2013, 135, 18536. 24199846 10.1021/ja4086758 PMC4102385

[42] P. E. Chen , N. C. Anderson , Z. M. Norman , J. S. Owen , J. Am. Chem. Soc. 2017, 139, 3227. 28125780 10.1021/jacs.6b13234

[43] S. Singh , R. Tomar , S. ten Brinck , J. De Roo , P. Geiregat , J. C. Martins , I. Infante , Z. Hens , J. Am. Chem. Soc. 2018, 140, 13292. 30253644 10.1021/jacs.8b07566

[44] M. Saniepay , C. Mi , Z. Liu , E. P. Abel , R. Beaulac , J. Am. Chem. Soc. 2018, 140, 1725. 29293359 10.1021/jacs.7b10649

[45] M.‐J. Choi , L. K. Sagar , B. Sun , M. Biondi , S. Lee , A. M. Najjariyan , L. Levina , F. P. García de Arquer , E. H. Sargent , Nano Lett. 2021, 21, 6057. 34250796 10.1021/acs.nanolett.1c01286

[46] L. Asor , J. Liu , S. Xiang , N. Tessler , A. I. Frenkel , U. Banin , Adv. Mater. 2023, 35, 2208332. 10.1002/adma.202208332 36398421

[47] F. Pietra , L. De Trizio , A. W. Hoekstra , N. Renaud , M. Prato , F. C. Grozema , P. J. Baesjou , R. Koole , L. Manna , A. J. Houtepen , ACS Nano 2016, 10, 4754. 27065247 10.1021/acsnano.6b01266

[48] A. Antanovich , A. W. Achtstein , A. Matsukovich , A. Prudnikau , P. Bhaskar , V. Gurin , M. Molinari , M. Artemyev , Nanoscale 2017, 9, 18042. 29131231 10.1039/c7nr05065h

[49] Z. Li , X. Peng , J. Am. Chem. Soc. 2011, 133, 6578. 21476585 10.1021/ja108145c

[50] D. W. Lucey , D. J. MacRae , M. Furis , Y. Sahoo , A. N. Cartwright , P. N. Prasad , Chem. Mater. 2005, 17, 3754.

[51] J. J. Calvin , T. M. Kaufman , A. B. Sedlak , M. F. Crook , A. P. Alivisatos , Nat. Commun. 2021, 12, 2663. 33976186 10.1038/s41467-021-22947-x PMC8113276

[52] L. C. Allen , J. Am. Chem. Soc. 1989, 111, 9003.

[53] A. Holownia , C. N. Apte , A. K. Yudin , Chem. Sci. 2021, 12, 5346. 34163766 10.1039/d1sc00077b PMC8179550

[54] P. Mosset , R. Grée , Synlett 2013, 24, 1142.

[55] R. Hayashi , G. R. Cook , Org. Lett. 2007, 9, 1311. 17346056 10.1021/ol070235g PMC2553353

[56] H. P. Andaraarachchi , M. J. Thompson , M. A. White , H.‐J. Fan , J. Vela , Chem. Mater. 2015, 27, 8021.

[57] A. Stelmakh , G. Marnieros , E. Schrader , G. Nedelcu , O. Hordiichuk , E. Rusanov , I. Cherniukh , D. Zindel , H. Grützmacher , M. V Kovalenko , J. Am. Chem. Soc. 2025, 147, 11446. 40123235 10.1021/jacs.5c01305 PMC11969539

[58] R. Karel Čapek , I. Moreels , K. Lambert , D. De Muynck , Q. Zhao , A. Van Tomme , F. Vanhaecke , Z. Hens , J. Phys. Chem. C 2010, 114, 6371.

[59] C. A. Leatherdale , W. K. Woo , F. V. Mikulec , M. G. Bawendi , J. Phys. Chem. B 2002, 106, 7619.

[60] B. M. McMurtry , K. Qian , J. K. Teglasi , A. K. Swarnakar , J. De Roo , J. S. Owen , Chem. Mater. 2020, 32, 4358.

[61] D. Franke , D. K. Harris , O. Chen , O. T. Bruns , J. A. Carr , M. W. B. Wilson , M. G. Bawendi , Nat. Commun. 2016, 7, 12749. 27834371 10.1038/ncomms12749 PMC5114595

[62] X. Peng , J. Wickham , A. P. Alivisatos , J. Am. Chem. Soc. 1998, 120, 5343.

[63] D. C. Gary , B. A. Glassy , B. M. Cossairt , Chem. Mater. 2014, 26, 1734.

[64] D. Franke , D. K. Harris , L. Xie , K. F. Jensen , M. G. Bawendi , Angew. Chem. 2015, 127, 14507. 10.1002/anie.201505972 26437711

[65] S. Tamang , S. Lee , H. Choi , S. Jeong , Chem. Mater. 2016, 28, 8119.

[66] Y. Li , X. Hou , Y. Shen , N. Dai , X. Peng , Chem. Mater. 2021, 33, 9348.

[67] O. B. Achorn , D. Franke , M. G. Bawendi , Chem. Mater. 2020, 32, 6532.

[68] R. Inoue , M. Yamaguchi , Y. Murakami , K. Okano , A. Mori , ACS Omega 2018, 3, 12703. 30411016 10.1021/acsomega.8b01707 PMC6210062

[69] M. Grabolle , M. Spieles , V. Lesnyak , N. Gaponik , A. Eychmüller , U. Resch‐Genger , Anal. Chem. 2009, 81, 6285.

[70] K. Rurack , M. Spieles , Anal. Chem. 2011, 83, 1232. 21250654 10.1021/ac101329h

[71] C. Morrison , H. Sun , Y. Yao , R. A. Loomis , W. E. Buhro , Chem. Mater. 2020, 32, 1760.

