# Supplementary material for: Tribenzoylarsine – A Benign Arsenic Precursor for InAs Nanocrystals
Source: Small. 2025 Sep 10;21(42):e04624. doi: 10.1002/smll.202504624 (PMC12547999; doi:10.1002/smll.202504624)
Supplement: Supplementary file 1 — Supporting Information [file SMLL-21-e04624-s001.pdf]

NANO · MICRO
small

Supporting Information

for *Small*, DOI 10.1002/smll.202504624

Tribenzoylarsine – A Benign Arsenic Precursor for InAs Nanocrystals

Artsiom Antanovich, Volodymyr Shamraienko, Jannika Lauth and Vladimir Lesnyak**

SUPPORTING INFORMATION

Tribenzoylarsine – an Arsenic Precursor for InAs Quantum Dots

Artsiom Antanovich,[1,2*] Volodymyr Shamraienko,[2] Jannika Lauth,[1,3-5] Vladimir Lesnyak[2*]

[1] Institute of Physical Chemistry and Electrochemistry, Leibniz University Hannover, Callinstraße 3a, 30167 Hannover, Germany

[2] Physical Chemistry, TU Dresden, Zellescher Weg 19, 01069 Dresden, Germany

[3] Laboratory of Nano and Quantum Engineering, Leibniz Universität Hannover, Schneiderberg 39, 30167, Hannover, Germany

[4] Cluster of Excellence PhoenixD (Photonics, Optics, and Engineering – Innovation Across Disciplines), Hannover, Welfengarten 1A, 30167, Germany

[5] Institute of Physical and Theoretical Chemistry, University of Tübingen, Auf der Morgenstelle 18, 72076, Tübingen, Germany

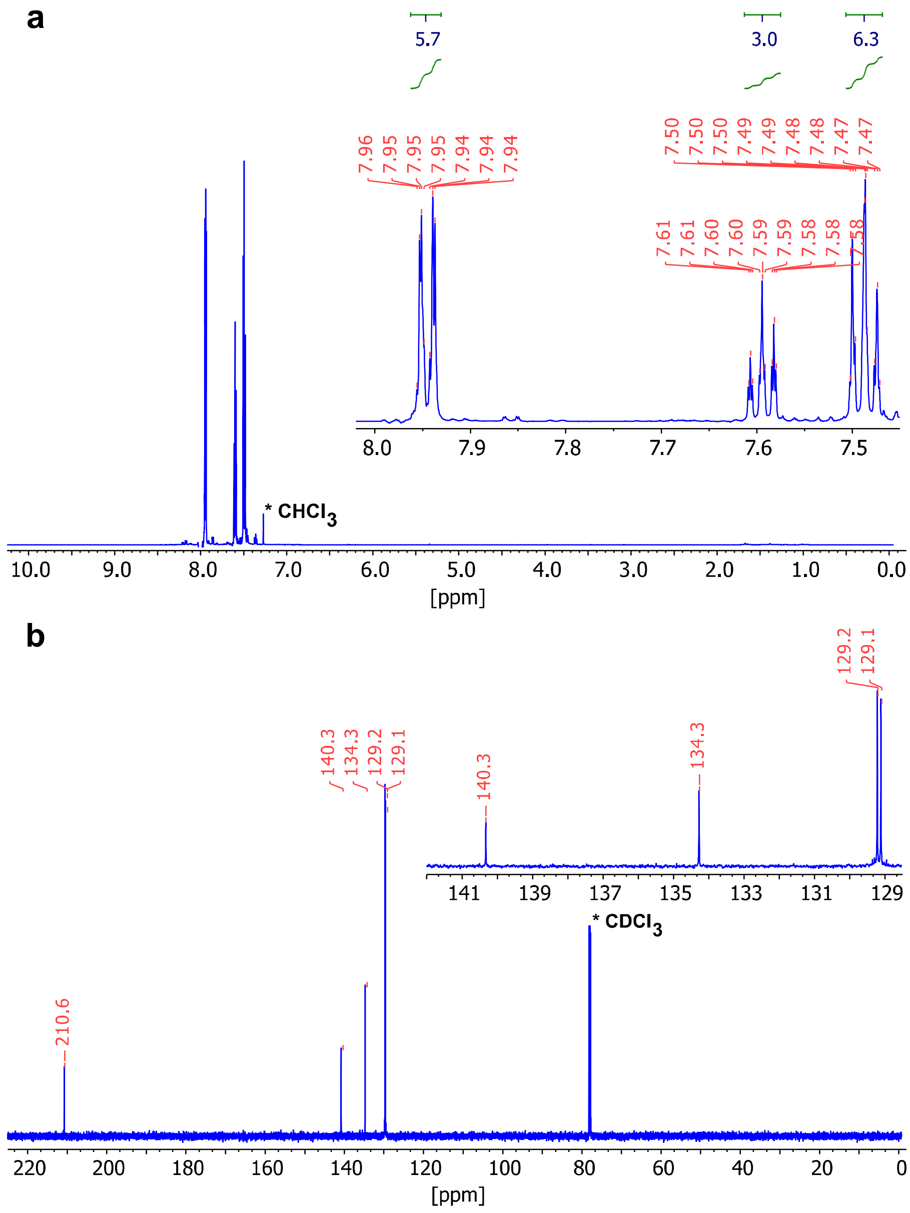

Figure S1. ^{1}H- (a) and ^{13}C-NMR (b) spectra of tribenzoylarsine in $CDCl_3$.

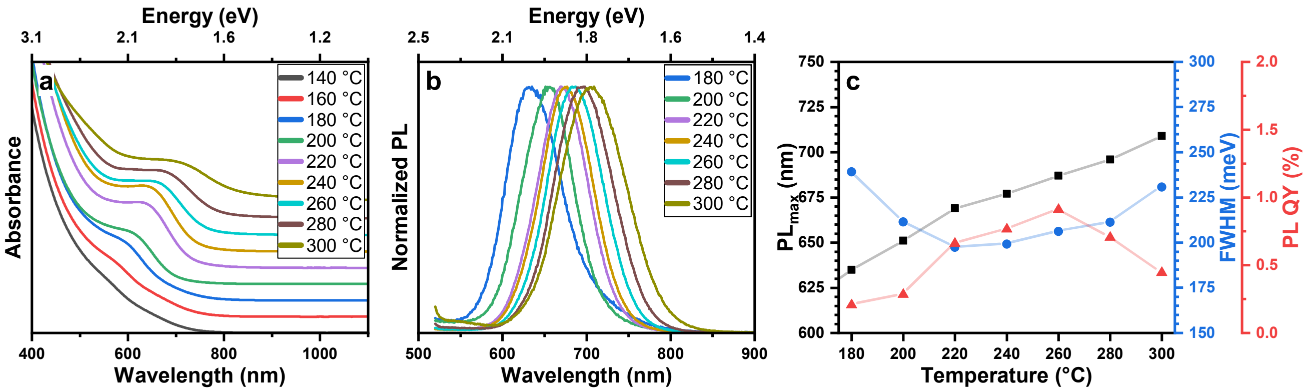

Figure S2. Absorption (a), PL (b) spectra and spectral parameters (c) of In(Zn)As QDs prepared by the heat-up process in the presence of 0.5 mmol zinc oleate.

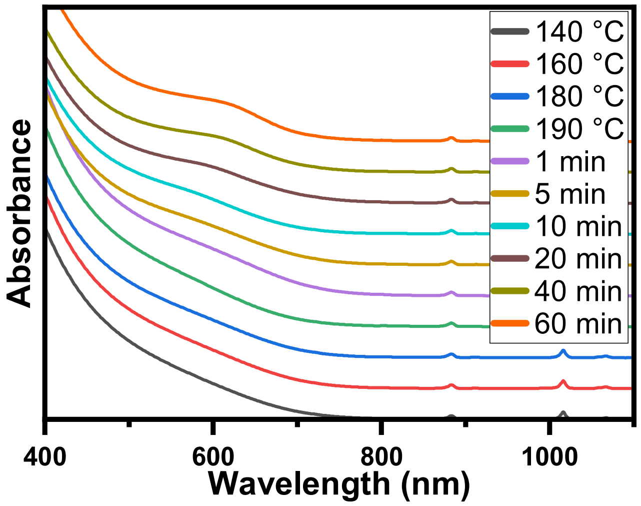

Figure S3. Evolution of the absorption spectra during the heat-up single-injection synthesis InAs QDs prepared using TMS_3As

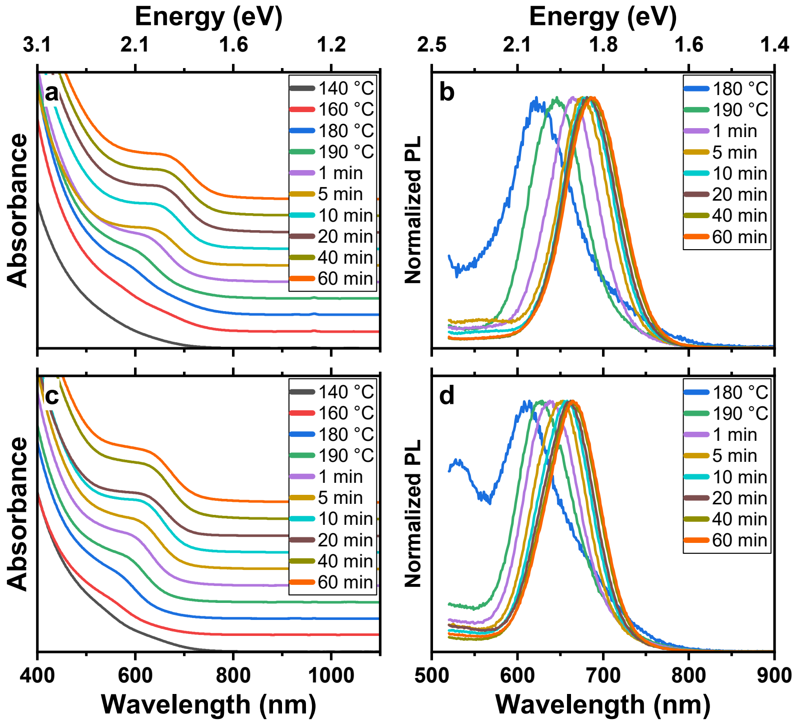

Figure S4. Evolution of the absorption (a, c) and PL (b, d) spectra during the heat-up single-injection synthesis In(Zn)As QDs prepared with the addition of 0.5 mmol (a,b) and 1.5 mmol (c,d) of zinc oleate.

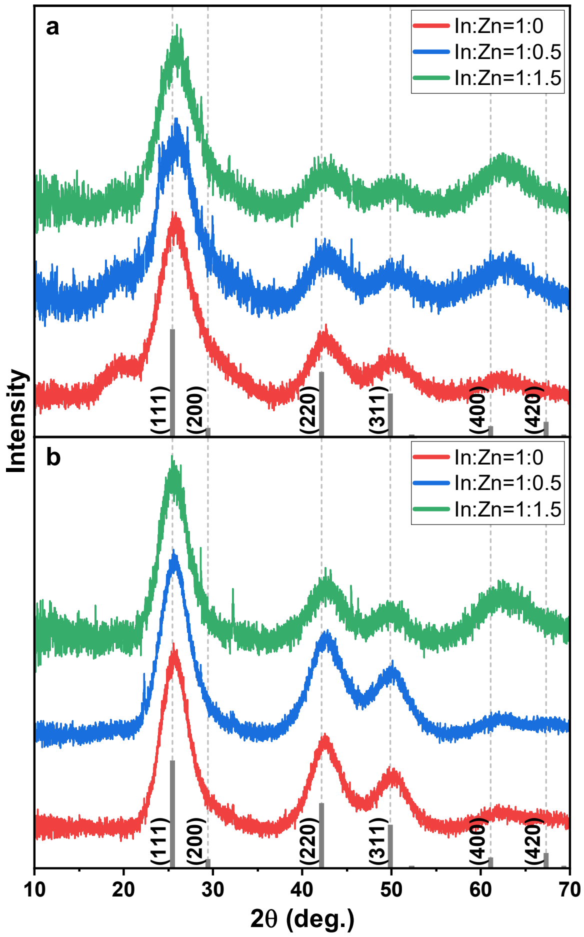

Figure S5. XRD patterns of In(Zn)As QDs prepared via injection of 0.4 mmol of Bz_3As followed by a slow injection of 0.2 mmol of Bz_3As (a) and via injection of 0.2 mmol of Bz_3As followed by a slow injection of 0.4 mmol of Bz_3As (b)

Table S1. Lattice constants and lattice constant changes relative to bulk InAs and InAs QDs prepared in Zn-free conditions.

Sample		a (Å)[1]	ε_{bulk} (%)	ε_{InAs} (%)
S04/02	In:Zn = 1:0	6.02	-0.7 %	-
	In:Zn = 1:0.5	5.99	-1.1 %	-0.4 %
	In:Zn = 1:1.5	6.02	-0.7 %	0 %
S02/04	In:Zn = 1:0	6.02	-0.7 %	-
	In:Zn = 1:0.5	6.02	-0.7 %	0 %
	In:Zn = 1:1.5	6.02	-0.7 %	0 %

[1] Average from positions of (111), (220) and (311) reflections.

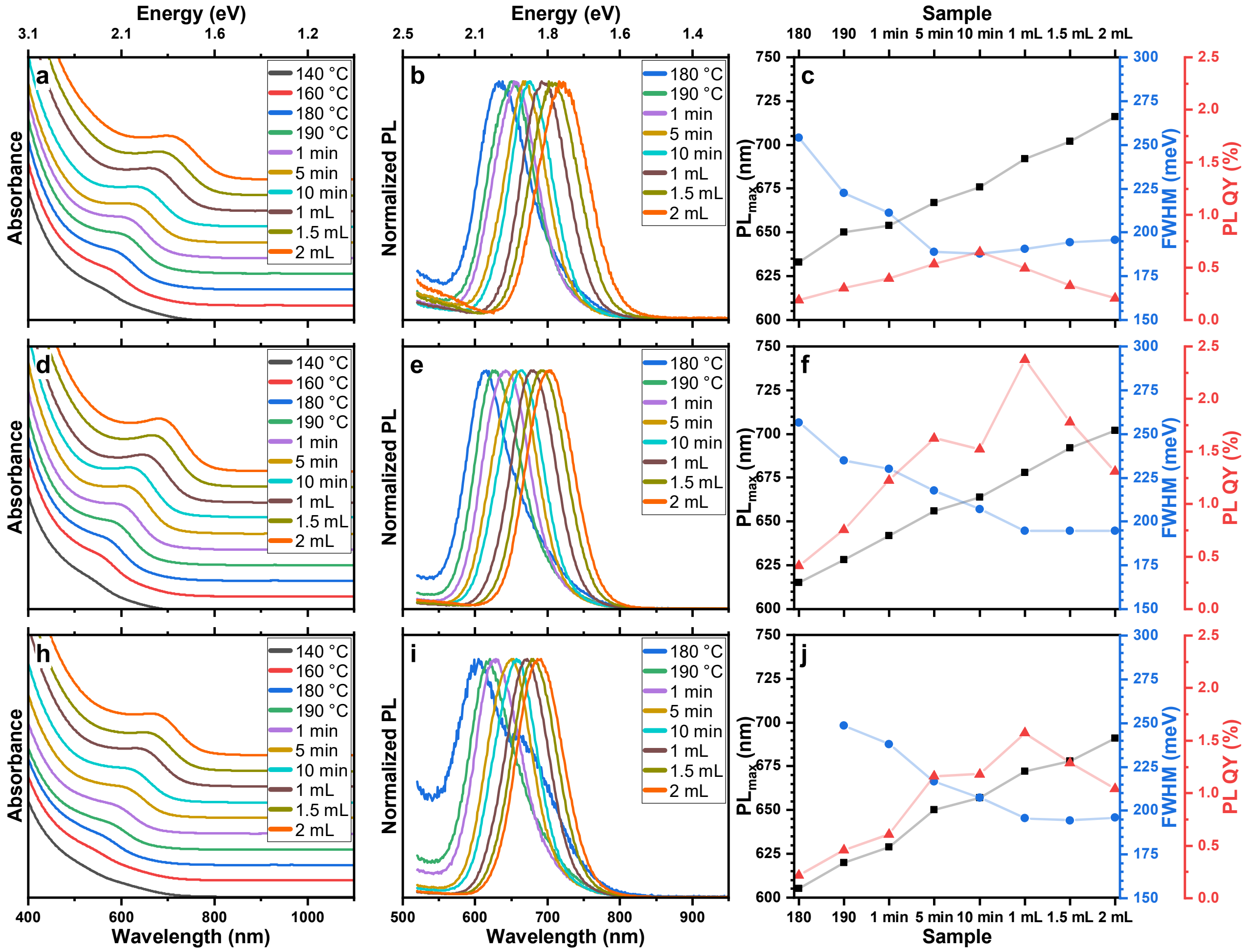

Figure S6. Evolution of the absorption (a, d, h), PL (b, e, i) spectra and spectral parameters (c, f, j) during the synthesis of In(Zn)As QDs prepared via injection of 0.4 mmol of Bz_3As followed by a slow injection of 0.2 mmol of Bz_3As without (a-c) and in the presence of 0.5 mmol (d-f) and 1.5 mmol (h-j) of zinc oleate.

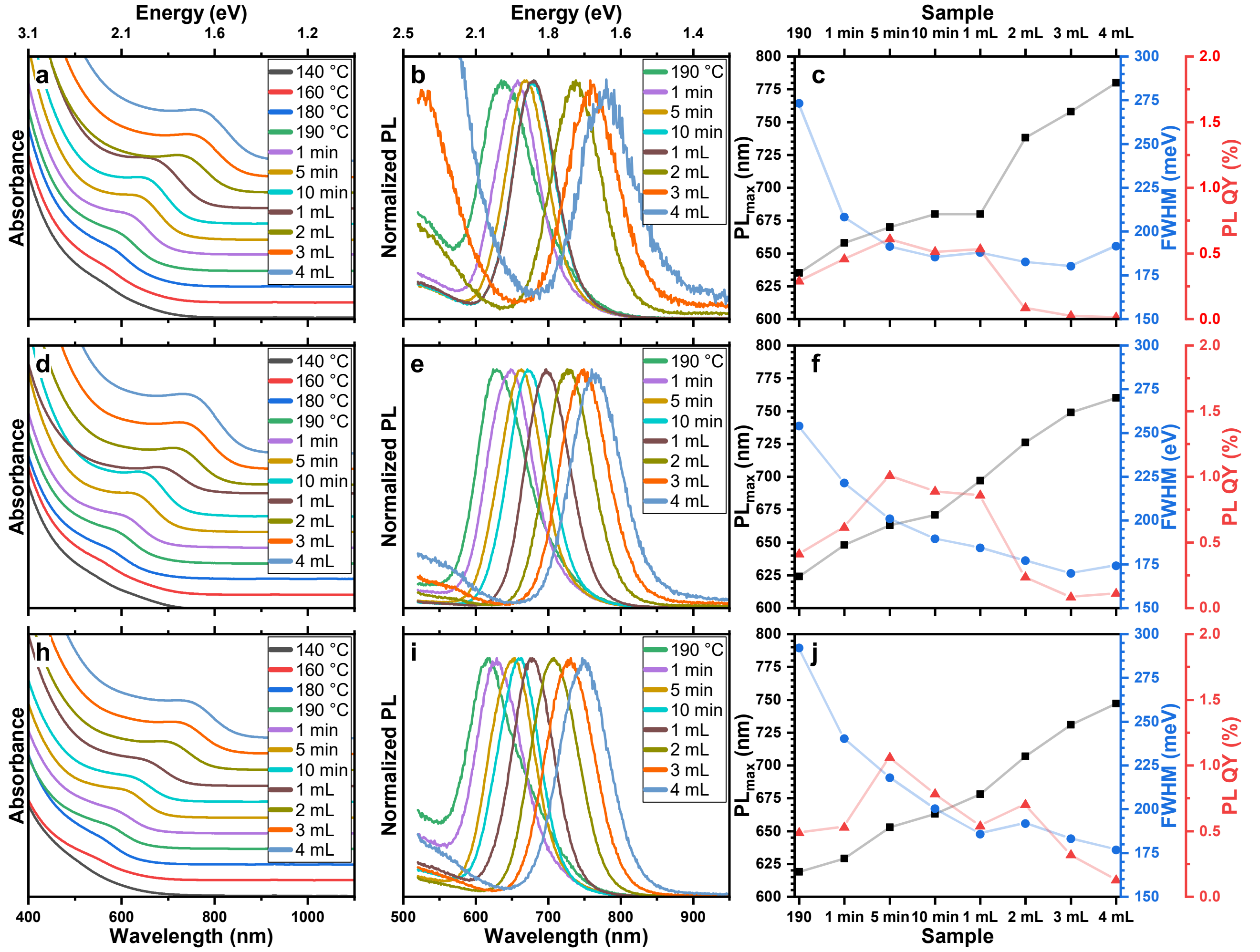

Figure S7. Evolution of the absorption (a, d, h), PL (b, e, i) spectra and spectral parameters (c, f, j) during the synthesis of In(Zn)As QDs prepared via injection of 0.2 mmol of Bz_3As followed by a slow injection of 0.4 mmol of Bz_3As without (a-c) and in the presence of 0.5 mmol (d-f) and 1.5 mmol (h-j) of zinc oleate.

Determination of the chemical yield

The chemical yield was estimated according to the methodology described in refs. [1,2], which relies on the assumption that the intrinsic absorption coefficient of nanocrystals at short wavelengths is very close to that of the bulk material.[3] At specified timepoints of the reaction ~ 150 μL of the reaction mixture were withdrawn from the reaction mixture. Then, an aliquot of a certain volume was taken out of this solution, diluted to 3 mL and measured.

Intrinsic absorption coefficient of indium arsenide was calculated according to the equation:[4]

$$\mu_i = \frac{4\pi nk|f_{LF}|^2}{n_s}$$

where n and k are the real and imaginary part of the refractive index of bulk zinc-blende InAs and n_s is the refractive index of the solvent.

$$|f_{LF}| = \frac{9n_s^4}{(n^2 - k^2 + 2n_s^2)^2 + 4(nk)^2}$$

using literature data, at 400 nm: n = 3.108,[5] k = 1.957[5] and n_s (chloroform) = 1.463[6], which yields $|f_{LF}| = 0.165$ and $\mu_i = 2.15 \cdot 10^5$ cm^{-1}.

Using these values, the volume fraction of InAs in the cuvette can be calculated from absorption as:

$$f_{cuvette} = \frac{A \cdot \ln(10)}{\mu_i \cdot L}$$

The total amount of InAs in the reaction mixture is:

$$n_{InAs}^{total} = \frac{\rho_{InAs} \cdot V_{cuvette} \cdot D}{M_{InAs}}$$

where $\rho_{InAs} = 5.667$ g/cm^3 is the density of InAs,[7] $V_{cuvette}$ is the volume of the solution in the cuvette; D is the dilution factor and $M_{InAs} = 189.74$ g/mol is the molar mass of InAs.

Finally, the reaction yield is determined by:

$$\varphi(InAs, \%) = \frac{n_{InAs}^{total}}{n_{Bz_3As}}$$

where n_{Bz_3As} is the amount of Bz_3As used for the synthesis (0.6 mmol).

References

[1] V. Grigel, D. Dupont, K. De Nolf, Z. Hens, M. D. Tessier, InAs Colloidal Quantum Dots Synthesis via Aminopnictogen Precursor Chemistry, *J. Am. Chem. Soc.* **2016**, *138*, 13485. https://doi.org/10.1021/jacs.6b07533.

[2] M. D. Tessier, D. Dupont, K. De Nolf, J. De Roo, Z. Hens, Economic and Size-Tunable Synthesis of InP/ZnE (E = S, Se) Colloidal Quantum Dots., *Chem. Mater.* **2015**, *27*, 4893. https://doi.org/10.1021/acs.chemmater.5b02138.

[3] C. A. Leatherdale, W. K. Woo, F. V. Mikulec, M. G. Bawendi, On the absorption cross section of CdSe nanocrystal quantum dots, *J. Phys. Chem. B* **2002**, *106*, 7619. https://doi.org/10.1021/jp025698c.

[4] R. Karel Čapek, I. Moreels, K. Lambert, D. De Muynck, Q. Zhao, A. Van Tomme, F. Vanhaecke, Z. Hens, Optical Properties of Zincblende Cadmium Selenide Quantum Dots, *J. Phys. Chem. C* **2010**, *114*, 6371. https://doi.org/10.1021/jp1001989.

[5] D. E. Aspnes, A. A. Studna, Dielectric functions and optical parameters of Si, Ge, GaP, GaAs, GaSb, InP, InAs, and InSb from 1.5 to 6.0 eV, *Phys. Rev. B* **1983**, *27*, 985. https://doi.org/10.1103/PhysRevB.27.985.

[6] A. Samoc, Dispersion of refractive properties of solvents: Chloroform, toluene, benzene, and carbon disulfide in ultraviolet, visible, and near-infrared, *J. Appl. Phys.* **2003**, *94*, 6167. https://doi.org/10.1063/1.1615294.

[7] O. Madelung, Semiconductors: Data Handbook, *Semiconductors: Data Handbook*, Springer Berlin Heidelberg, Berlin, Heidelberg, **2004**, . https://doi.org/10.1007/978-3-642-18865-7.
